# Ischaemic heart disease in the former Soviet Union 1990–2015 according to the Global Burden of Disease 2015 Study

**DOI:** 10.1136/heartjnl-2016-311142

**Published:** 2017-09-07

**Authors:** Adrianna Murphy, Catherine O Johnson, Gregory A Roth, Mohammad H Forouzanfar, Mohsen Naghavi, Marie Ng, Nana Pogosova, Theo Vos, Christopher J L Murray, Andrew E Moran

**Affiliations:** 1 Department of Health Services Research and Policy, London School of Hygiene and Tropical Medicine, London, UK; 2 Institute for Health Metrics and Evaluation, University of Washington, Seattle, Washington, USA; 3 Division of Cardiology, Department of Medicine, University of Washington, Seattle, Washington, USA; 4 National Research Centre for Preventive Medicine, Moscow, Russia; 5 Division of General Medicine, Department of Medicine, Columbia University Medical Center, New York, New York, USA

**Keywords:** Heart Disease, Epidemiology, Global Disease Patterns

## Abstract

**Objective:**

The objective of this study was to compare ischaemic heart disease (IHD) mortality and risk factor burden across former Soviet Union (fSU) and satellite countries and regions in 1990 and 2015.

**Methods:**

The fSU and satellite countries were grouped into Central Asian, Central European and Eastern European regions. IHD mortality data for men and women of any age were gathered from national vital registration, and age, sex, country, year-specific IHD mortality rates were estimated in an ensemble model. IHD morbidity and mortality burden attributable to risk factors was estimated by comparative risk assessment using population attributable fractions.

**Results:**

In 2015, age-standardised IHD death rates in Eastern European and Central Asian fSU countries were almost two times that of satellite states of Central Europe. Between 1990 and 2015, rates decreased substantially in Central Europe (men −43.5% (95% uncertainty interval −45.0%, −42.0%); women −42.9% (−44.0%, −41.0%)) but less in Eastern Europe (men −5.6% (−9.0, –3.0); women −12.2% (−15.5%, −9.0%)). Age-standardised IHD death rates also varied within regions: within Eastern Europe, rates decreased −51.7% in Estonian men (−54.0, −47.0) but increased +19.4% in Belarusian men (+12.0, +27.0). High blood pressure and cholesterol were leading risk factors for IHD burden, with smoking, body mass index, dietary factors and ambient air pollution also ranking high.

**Conclusions:**

Some fSU countries continue to experience a high IHD burden, while others have achieved remarkable reductions in IHD mortality. Control of blood pressure, cholesterol and smoking are IHD prevention priorities.

## Introduction

The fall of Communism from 1987 to 1994 had a calamitous effect on health in former Soviet Union (fSU) member and satellite countries. Life expectancy plummeted in fSU countries, increasing the health gap with the rest of Europe. Since the early 1990s, however, some fSU and former satellite countries have fared better than others in terms of all-cause mortality.^1^ For example, although life expectancy in former Soviet satellite countries of Central Europe had stagnated or declined in the late 1980s, it improved steadily following the collapse of the Berlin Wall. After 1998, life expectancy in the Baltic countries of the fSU improved consistently, both leading up to and after their joining the European Union in 2004.^2^

Ischaemic heart disease (IHD) was a leading contributor to the East-West health gap observed in Europe in the early 1990s.^3^ However, IHD burden across all the countries of the region in the years since 1990 has not been compared at the national level, using a standardised method. The Global Burden of Disease, Risk Factors, and Injuries 2015 Study (GBD 2015 Study)^4–7^ produced estimates of fatal and non-fatal IHD burden and IHD risk factors for each fSU and former satellite country in the years 1990 and 2015. We set out to compare 2015 IHD mortality across countries, change in IHD mortality between 1990 and 2015, and relative ranking of burden of disease attributable to IHD risk factors in fSU and satellite countries and the GBD regions including them (Central Asia, Central Europe, Eastern Europe).

## Methods

The fSU and satellite countries (ie, formally independent but heavily under Soviet control politically and economically) were defined as Armenia, Azerbaijan, Georgia, Kazakhstan, Kyrgyzstan, Mongolia, Tajikistan, Turkmenistan and Uzbekistan (Central Asian countries); Albania, Bulgaria, Czech Republic, Hungary, Poland, Romania and Slovakia (Central European countries); Belarus, Estonia, Latvia, Lithuania, Moldova, Russia and Ukraine (Eastern European countries). Bosnia and Herzegovina, Croatia, Macedonia, Montenegro, Serbia and Slovenia were included in this analysis as part of the Central Europe region.

GBD IHD burden estimation methods are described in detail in the online supplementary file 1 and in other published sources.^4–7^ The GBD 2015 Study estimated IHD deaths and burden. Burden is quantified in disability-adjusted life years (DALYs). DALYs are composed of years of life lost (YLL) to premature deaths (mortality) and years lived with non-fatal disease disability (years lost due to disability, morbidity). YLL were calculated in relation to hypothetical age-specific reference life expectancies (eg, 86.6 years at birth) derived from life table methods.

10.1136/heartjnl-2016-311142.supp1 Supplementary file 1

IHD has been consistently defined as an underlying cause of death across International Classification of Diseases (ICD) revisions (most recently ICD-10 I20-I25, ICD-9 410-414).^8^ The GBD systematically reallocated proportions of ill-defined or erroneously assigned deaths to IHD based on the total distribution of actual causes of death by the country, sex, age and year.^9^ Mortality data were mapped to GBD cause categories and distribution of deaths to IHD and other causes and regional mortality and temporal trends were estimated using the cause of death ensemble modelling (CODEm) approach (see online supplementary file 1).^10^ The cause of death correct (CoDCorrect) algorithm estimated total cardiovascular diseases as a proportion of all deaths, by which IHD deaths fit into the total cardiovascular disease mortality envelope. Because heart failure is not defined as an underlying cause of death in the ICD classification system, separate methods were developed in order to distribute heart failure deaths to IHD and other upstream causes of heart failure.^11^

Rates of non-fatal IHD prevalence, incidence and excess mortality (non-fatal myocardial infarction (MI), angina and ischaemic heart failure) were estimated from rates found in a systematic review of epidemiologic literature using a Bayesian metaregression method (DisMod-MR, see online supplementary file 1).^12^ The literature review of IHD epidemiology studies was first conducted in 2010 (for papers published between 1980 and 2008) and later updated to include all papers published betwen 2008 and 2015 (see online supplementary file 1). Papers identified in an all-world-regions electronic search for IHD epidemiology studies were initially selected if they reported on one of the epidemiologic parameters of interest (IHD death, acute MI, angina, or ischaemic heart failure incidence, prevalence, case fatality or mortality) and were population based. Fifty papers were included in the DisMod IHD model from the fSU and satellite state regions. Data for chronic IHD and post-MI survivors in Poland were added from the Prospective Rural and Epidemiological Study.^13^ In addition to published studies identified in the systematic review, the WHO World Health Survey provided data on angina prevalence, hospital admission registries and administrative data provided heart failure data, and unpublished IHD surveillance data for years following 1994 were provided from the WHO Multinational Monitoring of Trends and Determinants in Cardiovascular Disease (MONICA) surveillance site in Kaunas, Lithuania. Disability from each case of MI, angina, heart failure and other diseases or injuries was estimated in household surveys of laypeople in Bangladesh, Indonesia, Peru, Tanzania, the USA, Hungary, Italy, the Netherlands and Sweden, and an international web-based survey of health professionals.^14^ Angina symptom severity (mild, moderate or severe) was characterised according to the Canadian Cardiovascular Society classification^15^ and angina and heart failure symptom severity distributions were based on Short Form 12 quality of life scores in the US Medical Expenditure Panel Survey.

One thousand draws were taken of the posterior distributions of model covariate effects (beta coefficients), sampling error and data manipulations of CODEm and DisMod estimates, and model selection uncertainty of CODEm estimates. These uncertainty estimates reflect Bayesian credible intervals as opposed to CIs. Both approaches yield similar numerical results in large samples.^16^ Ninety-five per cent uncertainty intervals reported in the paper represent the 2.5 and 97.5 percentiles of these posterior distributions. IHD burden in the GBD 2015 Eastern Europe, Central Asia and Central Europe regions and at the country level is reported as age-standardised death rates and DALYs per 100 000 people. A GBD standard population^4^ was used to age standardise rates using the direct method. Age-standardised death rates for each year between 1990 and 2015 were also plotted.

The Comparative Risk Assessment arm of the GBD 2015 estimated the burden of IHD attributable to risk factors.^17^ National and subnational risk factor surveys were analysed in order to estimate the distribution of exposure to each risk factor. An optimal, theoretical minimum risk exposure level was selected and the relative risk per unit of risk factor exposure was obtained from meta-analyses. Minimum risk exposure levels for cardiometabolic risk factors were based on analysis of pooled prospective cohort studies and represent theoretical exposure levels at which risk is minimised (eg, systolic blood pressure (SBP) ~115 mm Hg) rather than the results of any known intervention that reduces risks. Attributable burden for every individual risk was calculated using the population attributable fraction method. The main risk factor clusters were hazardous alcohol consumption, tobacco consumption, low physical activity, physiological factors (high fasting plasma glucose, high total cholesterol, high blood pressure, high body mass index (BMI)), diet (diet low in fruits, diet low in vegetables, diet low in whole grains, diet low in nuts and seeds, diet high in processed meat, diet high in sugar-sweetened beverages, diet low in fibre, diet low in seafood omega-3 fatty acids, diet low in polyunsaturated fatty acids, diet high in trans fatty acids, diet high in sodium), air pollution (ambient particulate matter pollution or household air pollution from solid fuels) and other environmental risks (lead exposure). The total burden of risks under each cluster was calculated assuming independent effect of risks except for some risks for which the total burden was corrected by the amount of overlap and mediated effect through other risks (eg, some part of burden of elevated BMI was assumed to be mediated through elevated SBP).^7^ Smoking was defined as prevalence of daily tobacco smoking and number of cigarettes smoked per day, with a lag in effect applied. Country-level alcohol exposure was estimated by way of national level consumption (mean litres of alcohol consumed per capita annually) and the distribution of daily alcohol intake among alcohol drinkers. For the GBD 2015, the association of alcohol consumption with IHD included both an average daily intake effect and a binge/episodic drinking effect.^7^ Binge/episodic drinking pattern is more prevalent in countries of Eastern Europe and so a stronger adverse effect of alcohol, on a per capita basis, was estimated for those countries based on evidence from Russia.^18^ IHD risk factors were ranked in terms of attributable IHD morbidity burden (number of IHD DALYs).

## Results

**Figure 1:**
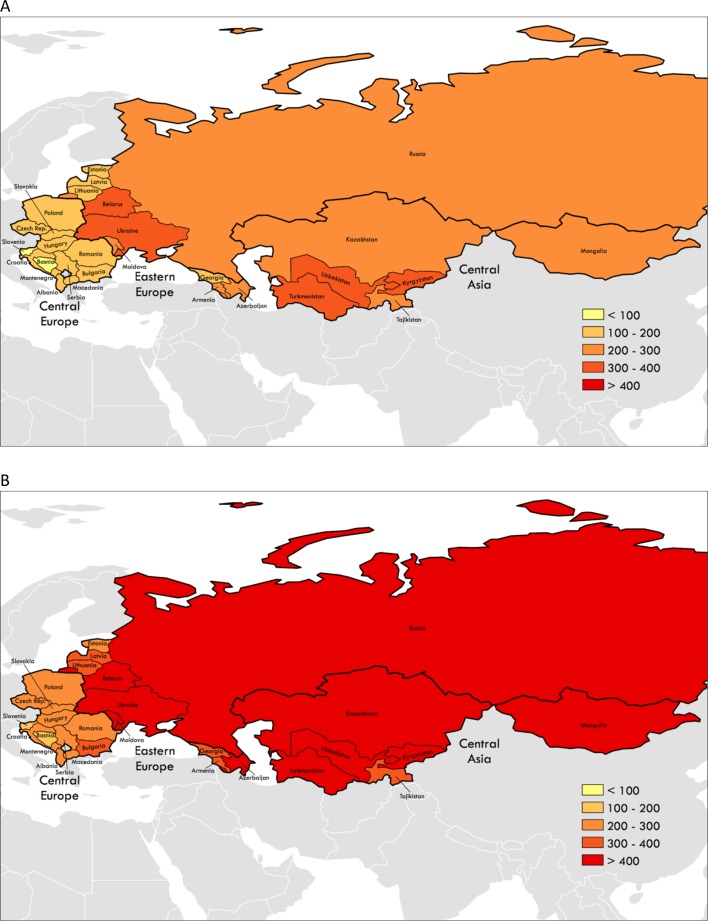
Age-standardised ischaemic heart disease mortality per 100 000 people, former Soviet Union and satellite state territories, 2015. (A) Women. (B) Men (produced using ArcGIS and Global Burden of Disease 2015 data).

In 2015, mean age-standardised IHD mortality per 100 000 men was almost twice as high in Eastern Europe (444.9) and Central Asia (425.2) compared with Central Europe (234.0). Among women, a similar pattern was observed, with rates almost twice as high in Eastern Europe (252.1) and Central Asia (271.1) as in Central Europe (141.3). Countries’ 2015 IHD burden varied within each region: age-standardised IHD death rates and IHD DALYs per 100 000 varied for both men and women among countries by at least 1.5 times in each region (comparing highest and lowest IHD burden countries within each of the three regions, figure 1, table 1, see online supplementary file 1).

**Table 1:** (A) Women, country level estimates of age-standardised IHD death rates per 100 000 people in 1990 and 2015, and per cent change over time, by country and region. (B) Men, country level estimates of age-standardised IHD death rates per 100 000 people in 1990 and 2015, and per cent change over time, by country and region

(A) Women	1990			2015			Median % change	95% UI	
	Mean	95% UI		Mean	95% UI				
**Central Asia**									
Armenia	297.0	276.5	317.0	201.5	184.2	221.4	−32.1%	−38.0%	−26.0%
Azerbaijan	459.9	439.0	479.2	268.2	250.5	286.5	−41.8%	−46.0%	−37.0%
Georgia	324.0	303.7	343.5	192.9	177.2	211.3	−40.6%	−47.0%	−33.0%
Kazakhstan	283.1	273.9	291.1	267.5	245.5	290.5	−5.5%	−14.0%	3.0%
Kyrgyzstan	278.7	262.7	296.1	308.0	287.3	327.6	10.7%	1.0%	21.0%
Mongolia	315.2	280.1	352.9	259.7	232.7	293.9	−17.9%	−29.0%	−5.0%
Tajikistan	311.6	292.5	329.8	277.6	256.6	302.1	−11.1%	−18.0%	−3.0%
Turkmenistan	388.4	377.4	399.5	332.5	316.8	349.7	−14.6%	−19.0%	−10.0%
Uzbekistan	350.1	327.2	374.1	308.4	276.3	333.3	−11.7%	−22.0%	−3.0%
**All**	324.9	316.5	332.6	271.1	259.2	282.1	−16.5%	−20.0%	−13.0%
**Central Europe**									
Albania	128.4	119.1	140.0	126.1	113.8	139.3	−1.7%	−15.0%	11.0%
Bosnia and Herzegovina	196.1	181.3	211.0	88.5	77.5	100.5	−54.9%	−61.0%	−47.0%
Bulgaria	283.8	275.5	292.5	188.8	178.1	199.9	−33.6%	−38.0%	−29.0%
Croatia	186.5	179.2	194.2	139.1	131.2	146.8	−25.4%	−30.0%	−20.0%
Czech Republic*	257.3	249.1	265.7	146.7	140.0	153.1	−43.0%	−46.0%	−40.0%
Hungary	227.6	220.7	234.7	155.4	147.2	164.5	−31.8%	−36.0%	−27.0%
Macedonia	154.7	142.8	167.2	117.2	105.6	129.8	−24.2%	−33.0%	−14.0%
Montenegro	129.4	113.0	146.4	112.1	98.5	126.3	−13.3%	−28.0%	4.0%
Poland	271.8	265.0	279.0	121.1	115.5	127.0	−55.4%	−58.0%	−53.0%
Romania	249.2	241.8	256.6	161.0	152.2	170.6	−35.4%	−39.0%	−31.0%
Serbia	196.6	181.3	214.4	141.0	131.7	151.5	−28.0%	−36.0%	−21.0%
Slovakia	306.7	294.7	317.4	174.4	165.8	183.1	−43.2%	−46.0%	−40.0%
Slovenia	126.1	119.4	132.8	67.4	62.1	72.6	−46.6%	−51.0%	−42.0%
**All**	247.3	243.5	251.4	141.3	137.7	144.9	−42.9%	−44.0%	−41.0%
**Eastern Europe**									
Belarus	315.1	303.1	326.7	309.6	292.1	326.6	−1.8%	−8.0%	5.0%
Estonia	301.4	293.3	310.2	153.2	141.5	163.9	−49.1%	−53.0%	−45.0%
Latvia	289.0	279.6	298.4	177.7	169.2	187.0	−38.5%	−42.0%	−35.0%
Lithuania	307.5	300.7	314.1	181.3	174.4	188.2	−41.0%	−43.0%	−38.0%
Moldova	384.2	374.8	393.3	276.8	266.0	288.3	−28.0%	−31.0%	−25.0%
Russia	270.0	262.6	277.8	221.3	212.0	231.1	−18.1%	−22.0%	−14.0%
Ukraine	317.9	310.3	325.2	340.5	324.7	356.5	7.1%	2.0%	13.0%
**All**	287.2	281.4	292.9	252.1	244.6	259.8	−12.2%	−15.0%	−9.0%

(B) Men	1990			2015			Median % change	95% UI	
	Mean	95% UI		Mean	95% UI				
**Central Asia**									
Armenia	430.7	409.5	452.5	348.1	325.7	370.8	−19.2%	−25.0%	−13.0%
Azerbaijan	645.9	619.6	672.0	415.4	375.4	465.4	−36.0%	−42.0%	−28.0%
Georgia	511.7	477.3	548.6	389.9	354.0	420.4	−23.8%	−32.0%	−16.0%
Kazakhstan	451.7	438.9	463.6	435.5	400.3	471.2	−3.6%	−12.0%	4.0%
Kyrgyzstan	421.3	399.3	444.5	468.3	440.0	500.2	11.2%	3.0%	20.0%
Mongolia	380.7	300.1	418.1	417.7	378.8	452.7	9.2%	−1.0%	27.0%
Tajikistan	413.7	388.2	460.2	338.0	305.5	395.8	−18.3%	−25.0%	−10.0%
Turkmenistan	584.4	520.4	599.6	527.5	493.5	556.3	−9.8%	−14.0%	−5.0%
Uzbekistan	478.3	448.5	510.4	452.1	419.8	486.1	−5.3%	−13.0%	3.0%
**All**	482.3	470.3	494.2	425.2	408.6	442.4	−11.9%	−15.0%	−8.0%
**Central Europe**									
Albania	232.6	216.0	247.0	209.6	180.3	239.2	−9.6%	−23.0%	4.0%
Bosnia and Herzegovina	321.0	296.0	342.6	145.3	128.3	170.5	−54.9%	−60.0%	−49.0%
Bulgaria	414.4	402.0	425.5	313.0	298.8	328.9	−24.6%	−28.0%	−20.0%
Croatia	327.0	316.5	339.1	201.8	192.0	211.7	−38.3%	−42.0%	−35.0%
Czech Republic	464.1	452.8	476.0	226.2	217.8	234.9	−51.2%	−53.0%	−49.0%
Hungary	406.3	395.3	416.9	243.0	229.6	255.6	−40.2%	−44.0%	−37.0%
Macedonia	256.6	237.7	275.3	201.6	180.8	222.4	−21.4%	−30.0%	−13.0%
Montenegro	239.8	216.3	265.4	227.3	202.5	253.2	−5.0%	−18.0%	10.0%
Poland	505.0	494.1	515.3	223.5	215.1	232.0	−55.7%	−58.0%	−54.0%
Romania	369.0	357.2	378.3	267.5	253.9	281.8	−27.5%	−31.0%	−23.0%
Serbia	290.7	265.1	313.1	209.3	195.3	222.3	−27.8%	−35.0%	−20.0%
Slovakia	512.7	494.1	530.2	268.4	254.1	280.9	−47.5%	−51.0%	−45.0%
Slovenia	226.0	216.2	236.4	107.1	99.2	115.2	−52.7%	−56.0%	−49.0%
**All**	414.3	407.5	419.4	234.0	228.9	239.5	−43.5%	−45.0%	−42.0%
**Eastern Europe**									
Belarus	498.3	475.6	515.2	595.0	557.8	627.4	19.4%	12.0%	27.0%
Estonia	538.5	525.2	551.5	261.1	245.5	287.3	−51.7%	−54.0%	−47.0%
Latvia	515.6	502.4	528.3	325.3	312.1	341.1	−37.0%	−40.0%	−34.0%
Lithuania	480.6	471.3	489.9	336.3	324.9	351.2	−30.1%	−32.0%	−27.0%
Moldova	500.8	489.1	511.0	404.3	391.1	418.3	−19.2%	−22.0%	−16.0%
Russia	458.8	446.3	473.0	405.3	390.7	419.6	−11.6%	−16.0%	−8.0%
Ukraine	480.8	467.8	493.0	545.9	523.7	567.7	13.5%	9.0%	19.0%
**All**	471.8	461.5	482.4	444.9	433.3	456.5	−5.6%	−9.0%	−3.0%

IHD, ischaemic heart disease; UI, uncertainty interval.

Between 1990 and 2015, IHD death rates decreased most steeply in Central European men (median per cent change −43.5 (95% uncertainty interval −45.0, –42.0)) and women (−42.9 (−44.0, −41.0)) and proportionally less in Eastern European men (−5.6% (−9.0%, −3.0%)) and women (−12.2% (−15.0, –9.0)) and Central Asian men (−11.9% (−15.0, –8.0)) and women (−16.5% (−20.0, −13.0)).

**Figure 2:**
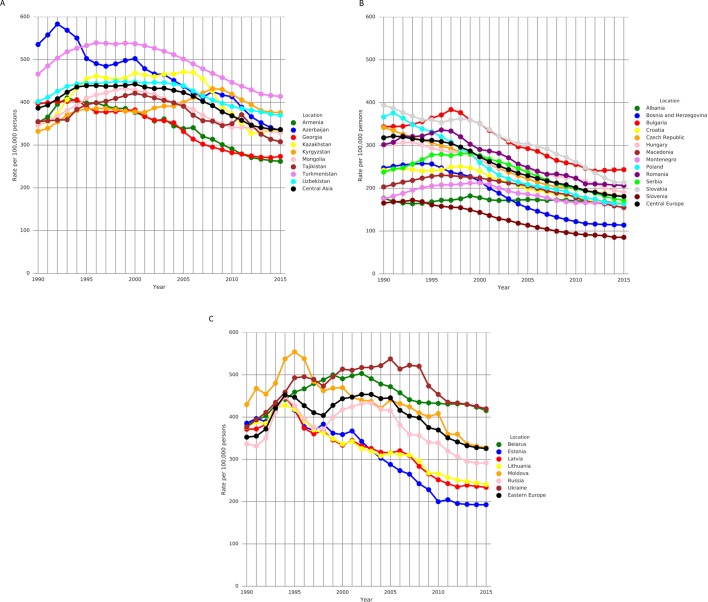
Annual country-level age-standardised ischaemic heart disease mortality rate per 100 000 people trends by region, men and women, 1990–2015. (A) Eastern Europe. (B) Central Asia. (C) Central Europe. Black line in each panel represents regional average (produced using R and Global Burden of Disease 2015 data).

At the national level, mortality trends varied among the fSU and satellite countries in 5-year periods over the interval 1990–2015 (figure 2). In Eastern Europe, age-standardised IHD mortality rates increased in all fSU countries in the early 1990s, after the fall of the Soviet Union. There was significant heterogeneity in overall change in age-standardised IHD death rate over time among countries within each region between 1990 and 2015 (table 1). Within Eastern Europe, male IHD death rates decreased markedly in Estonia (median per cent change −51.7% (−54.0, –47.0)) and Latvia (−37.0% (−40.0, −34.0)), with decreases in all other Eastern European countries except for Belarus and Ukraine, which experienced large increases (+19.4% (+12.0, +27.0) and +13.5% (9.0, 19.0) respectively). In Central Asia, the male IHD death rate decreased in all but Kyrgyzstan and Mongolia. The largest relative declines in male country-level age-standardised IHD death rate were observed in Central Europe; Central Europe and Estonia saw the largest declines in female rates. Age-standardised per capita IHD DALY decreases were seen in every country except for Belarus, Ukraine, Kyrgyzstan and Mongolia (for men) and Ukraine and Kyrgyzstan (for women) (online supplementary file 1).

**Figure 3:**
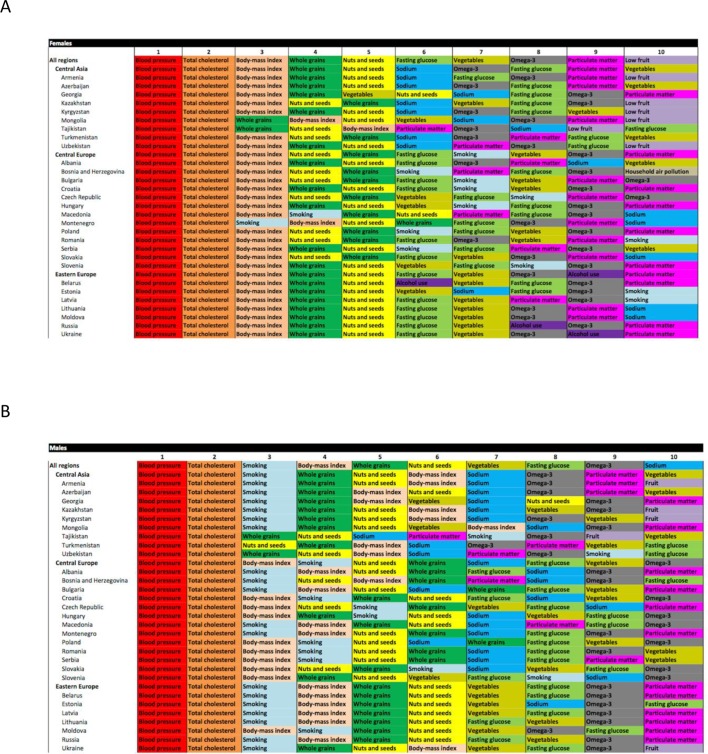
GBD 2015 Study ‘heat maps’ of risk factors ranked by attributable ischaemic heart disease disability-adjusted life years, 2015, age standardised, for men and women. (A) Women. (B) Men (produced using Excel and GBD 2015 data). GBD, Global Burden of Disease.

Relative rank of IHD burden (DALYs) attributable to major risk factors varied among countries of Central Europe, Eastern Europe and Central Asia in 2015 (figure 3). For men and women in all regions, high blood pressure was the leading risk factor contributing to IHD DALYs, with high total cholesterol the second leading risk factor. Depending on the country, BMI or smoking was the third leading risk factor. Hazardous alcohol consumption did not rank in the top 10 risk factors in any of the three fSU regions when men of all ages were combined. For women, it was the sixth leading risk factor in Belarus, the eighth in Russia and the ninth in Ukraine, and in the Eastern European region as a whole. However, taking younger adults (ages 15–49 years) alone, alcohol use was the seventh leading risk factor for male IHD DALYs in Belarus, Russia and Ukraine, and the eighth in Moldova, and for women alcohol ranked third in Belarus, fourth in Russia and Ukraine, and fifth in Moldova.^19^ Particulate matter (ambient air pollution) was among the 10 leading contributors to IHD DALYs for men and women in many countries, with dietary risk factors occupying the remaining leading risk factor positions.

## Discussion

While age-standardised IHD mortality rates fluctuated in fSU countries since 1990, most republics and satellite states experienced overall improvements in IHD mortality and morbidity since the breakup of the Soviet Union. In others, namely Belarus, Ukraine, Kyrgyzstan and Mongolia, per capita IHD burden increased. Despite recent favourable trends, countries of the fSU still experience IHD mortality rates almost twice as high as those of Central Europe, mirroring higher all-cause mortality compared with the rest of Europe.^2^ In the years following the fSU collapse, inflation rose, and by 1998 Russia suffered an economic crisis that spread to neighbouring countries and reversed health gains won between 1991 and1994. The Baltic Republics were less affected by the 1998 crisis and health indicators in these countries continued to improve after it.^2^ Low socioeconomic status and unstable employment,^1^ unrewarding employment,^20^ lack of social capital,^1 21^ community disengagement^22^ and widespread feelings of alienation and low personal control^23^ have all been linked to poor health in the fSU. In Russia, depression has been directly linked to unfavourable cardiovascular disease risk factors, and mood symptoms were strong predictors of mortality among patients with arterial hypertension and IHD.^24^

The overall pattern of IHD mortality that we observed in fSU countries between 1990 and 2015 is similar to that observed for life expectancy at birth for these countries (life expectancy source: WHO Health for All Database; online supplementary file 1). For example, in Eastern Europe, there was a large drop in life expectancy in all countries in the region in 1994, which corresponds to increases in IHD mortality in those countries that year. In Russia, IHD mortality began to increase again in 1998 and only fell to pre-1998 levels in 2006, which again corresponds to the pattern observed for life expectancy in that country.

The leading more proximal cause of high IHD burden in the fSU and satellite countries was uncontrolled high blood pressure. While high alcohol and dietary salt consumption and low consumption of fresh fruits and vegetables may contribute to high blood pressure, there is also broad variation in the capacity of national health systems to detect, treat and control hypertension in clinical practice. In the years since the fSU breakup, former member countries have engaged in health system reforms to varying degrees (some of these reforms more successful than others); however, state funding for healthcare remains almost universally insufficient and patient out-of-pocket (OOP) payments (formal and informal) may act as one barrier to accessing medicines for controlling hypertension and chronic IHD.^25 26^ Of adults in the region prescribed treatment for hypertension, almost two-thirds report not taking treatment daily.^27^ To effectively provide services and protect patients from catastrophic expenditure, it is recommended that public (or government) health expenditure as a proportion of GDP be at least 5%,^28^ and private (or OOP) expenditure on health occupy less than 15% of a country’s total health expenditure.^29^ Very few of the fSU countries included in our analysis meet these standards—for example, in Belarus, in 2014 public health expenditure was only 4% of GDP and OOP expenditure occupied 32% of total health expenditure; in Kyrgyzstan these figures are 4% and 39%, in Ukraine 4% and 46%. In the Czech Republic on the other hand, where an almost 50% reduction in male IHD mortality was observed, these figures are 6% and 14%.^30^

Hazardous alcohol consumption has been frequently implicated as a cause of high IHD mortality in Eastern Europe, particularly in Russia.^31^ The episodic heavy drinking pattern, sometimes referred to as ‘binge’ drinking, is relatively common in fSU countries,^21^ and is linked to increased risk of IHD mortality and morbidity.^32^ Hazardous alcohol consumption was an important IHD risk factor in Eastern European younger adults (aged 19–49 years) in our analysis. Nonetheless, the underlying mechanisms responsible for the link between alcohol and IHD deaths (or deaths classified as IHD) in the fSU remain controversial, and evidence suggests that this link contributes to most temporal *fluctuations* in mortality, but is not necessarily the main driver of an underlying steady state of high IHD mortality.^33^ While it appears that hazardous alcohol consumption is associated with the non-MI portion of IHD deaths, it remains unclear if this association is mediated by atherosclerotic plaque rupture in a coronary artery, alcohol-induced arrhythmias or alcohol–related cardiomyopathy,^34^ and how much of the association is confounded by tobacco use in heavy alcohol consumers.^35^ Misclassification of alcohol-related deaths as IHD deaths might overestimate the number of IHD deaths, resulting in inaccurately high estimated IHD mortality rates in countries with high per capita alcohol consumption.

### Limitations

For a number of countries analysed, especially the countries of Central Asia, IHD epidemiological data were limited. Very few of the studies providing data on non-fatal burden of IHD in the fSU and satellite states included women. For example, only a fifth of papers (n=14) identified in initial screening for the Eastern Europe IHD literature review collected or analysed data on women in their analyses. The under-representation of women in epidemiological studies in the fSU is especially problematic given the large variation in IHD risk factor behaviour (ie, drinking and smoking) between women of different fSU countries, and between women and men in this region.^36^ Our conclusions regarding IHD risk factors are useful for guiding health policy but do not represent the impact of interventions to address these risks. Further research is needed to identify which interventions will provide most health benefit and societal value in reducing attributable burden for each risk factor.

## Conclusions

Our analysis shows substantial variations in IHD mortality among the fSU and satellite countries from 1990 to 2015. The markedly different age-standardised IHD mortality trends in these countries call for a greater focus on interventions targeting IHD risk factors, but also the underlying social mechanisms contributing to the IHD epidemic in the region. Reduced IHD rates achieved in neighbouring Central European and Baltic Republic countries could serve as road maps for fSU countries that are seeking to reverse the epidemic of IHD.

**Key messages**

**What is already known on this subject?**

Following the fall of the Soviet Union in 1991, age-adjusted all-cause mortality was substantially higher in the former Soviet Union (fSU) and its former satellite states than in the rest of Europe. Subsequently, the health gap between some fSU or satellite states and the rest of Europe narrowed, but for other countries the gap persisted. Past research implicated ischaemic heart disease (IHD) as an important contributor to the East-West Europe health gap in the early 1990s, and focused predominantly on hazardous alcohol consumption and smoking as causes.

**What might this study add?**

We examined patterns in IHD mortality in the fSU from 1990 to 2015 and the contribution of risk factors to IHD burden. Our results show that death rates in Eastern European and Central Asian fSU countries in 2015 were almost two times that of the former satellite states of Central Europe. Between 1990 and 2015, the relative median decrease in male age-standardised IHD death rates in Central Europe was −43.5%, compared with only −5.6% in Eastern Europe. Temporal IHD mortality trends varied among countries within regions. High blood pressure and high cholesterol rank as the first and second-leading risk factors responsible for IHD burden in the fSU, but smoking, dietary and ambient air pollution also rank in the top 10.

**How might this impact on clinical practice?**

Our results indicate that clinical practice should prioritise treatment and control of traditional IHD risk factors like high blood pressure, high cholesterol and smoking in fSU countries. Controlling the IHD epidemic in the fSU will also require public health measures to limit the harms of ambient air pollution and hazardous alcohol consumption.

## References

[1] Carlson P The european health divide: a matter of financial or social capital? Soc Sci Med 2004;59:1985–92. 10.1016/j.socscimed.2004.03.003 15312932

[2] Leon DA Trends in european life expectancy: a salutary view. Int J Epidemiol 2011;40:271–7. 10.1093/ije/dyr061 21415000

[3] Zatonski W Closing the health gap in european union, 2008.

[4] Mortality GBD; GBD 2015 Mortality and Causes of Death Collaborators. Global, regional, and national life expectancy, all-cause mortality, and cause-specific mortality for 249 causes of death, 1980-2015: a systematic analysis for the global burden of disease study 2015. Lancet 2016;388:1459–544. 10.1016/S0140-6736(16)31012-1 27733281 PMC5388903

[5] GBD 2015 Disease and Injury Incidence and Prevalence Collaborators. Global, regional, and national incidence, prevalence, and years lived with disability for 310 diseases and injuries, 1990-2015: a systematic analysis for the global burden of disease study 2015. Lancet 2016;388:1545–602. 10.1016/S0140-6736(16)31678-6 27733282 PMC5055577

[6] GBD 2015 DALYs and HALE Collaborators. Global, regional, and national disability-adjusted life-years (DALYs) for 315 diseases and injuries and healthy life expectancy (HALE), 1990-2015: a systematic analysis for the global burden of disease study 2015. Lancet 2016;388:1603–58. 10.1016/S0140-6736(16)31460-X 27733283 PMC5388857

[7] Collaborators GGBDRF; GBD 2015 Risk Factors Collaborators. Global, regional, and national comparative risk assessment of 79 behavioural, environmental and occupational, and metabolic risks or clusters of risks, 1990-2015: a systematic analysis for the global burden of disease study 2015. Lancet 2016;388:1659–724. 10.1016/S0140-6736(16)31679-8 27733284 PMC5388856

[8] Moran AE, Oliver JT, Mirzaie M, et al Assessing the global burden of ischemic Heart disease: part 1: methods for a systematic review of the global epidemiology of ischemic Heart Disease in 1990 and 2010. Glob Heart 2012;7:315–29. 10.1016/j.gheart.2012.10.004 23682350 PMC3652434

[9] Naghavi M, Makela S, Foreman K, et al Algorithms for enhancing public health utility of national causes-of-death data. Popul Health Metr 2010;8:9 10.1186/1478-7954-8-9 20459720 PMC2873308

[10] Foreman KJ, Lozano R, Lopez AD, et al Modeling causes of death: an integrated approach using CODEm. Popul Health Metr 2012;10:10:1 10.1186/1478-7954-10-1 22226226 PMC3315398

[11] Forouzanfar MH, Moran AE, Flaxman AD, et al Assessing the global burden of ischemic heart disease, part 2: analytic methods and estimates of the global epidemiology of ischemic heart disease in 2010. Glob Heart 2012;7:331–42. 10.1016/j.gheart.2012.10.003 23505617 PMC3595103

[12] Moran AE, Forouzanfar MH, Roth GA, et al The global burden of ischemic heart disease in 1990 and 2010: the global burden of disease 2010 study. Circulation 2014;129:1493–501. 10.1161/CIRCULATIONAHA.113.004046 24573351 PMC4181601

[13] Corsi DJ, Subramanian SV, Chow CK, et al Prospective Urban Rural Epidemiology (PURE) study: Baseline characteristics of the household sample and comparative analyses with national data in 17 countries. Am Heart J 2013;166:636–46. 10.1016/j.ahj.2013.04.019 24093842

[14] Salomon JA, Vos T, Hogan DR, et al Common values in assessing health outcomes from disease and injury: disability weights measurement study for the global burden of disease study 2010. Lancet 2012;380:2129–43. 10.1016/S0140-6736(12)61680-8 23245605 PMC10782811

[15] Hemingway H, Fitzpatrick NK, Gnani S, et al Prospective validity of measuring angina severity with Canadian Cardiovascular Society class: the ACRE study. Can J Cardiol 2004;20:305–9. 15054509

[16] Bland JM, Altman DG Bayesians and frequentists. BMJ 1998;317:1151–60. 9784463 10.1136/bmj.317.7166.1151 PMC1114120

[17] Forouzanfar MH, Alexander L, Anderson HR, et al Global, regional, and national comparative risk assessment of 79 behavioural, environmental and occupational, and metabolic risks or clusters of risks in 188 countries, 1990-2013: a systematic analysis for the global burden of disease study 2013. Lancet 2015;386:2287–323. 10.1016/S0140-6736(15)00128-2 26364544 PMC4685753

[18] Zaridze D, Lewington S, Boroda A, et al Alcohol and mortality in Russia: prospective observational study of 151,000 adults. Lancet 2014;383:1465–73. 10.1016/S0140-6736(13)62247-3 24486187 PMC4007591

[19] Institute of Health Metrics and Evaluation. 19Gbd compare data visualizations: daly risk factor rankings. 2016;2016.

[20] Bobak M, Pikhart H, Kubinova R, et al The association between psychosocial characteristics at work and problem drinking: a cross-sectional study of men in three eastern european urban populations. Occup Environ Med 2005;62:546–50. 10.1136/oem.2004.017202 16046607 PMC1741061

[21] Murphy A, Roberts B, Kenward MG, et al Using multi-level data to estimate the effect of social capital on hazardous alcohol consumption in the former Soviet Union. Eur J Public Health 2014;24:572–7. 10.1093/eurpub/ckt213 24473595

[22] Palosuo H Health-related lifestyles and alienation in Moscow and Helsinki. Soc Sci Med 2000;51:1325–41. 10.1016/S0277-9536(00)00095-2 11037220

[23] Cockerham WC, Hinote BP, Abbott P Psychological distress, gender, and health lifestyles in Belarus, Kazakhstan, Russia, and Ukraine. Soc Sci Med 2006;63:2381–94. 10.1016/j.socscimed.2006.06.001 16887246

[24] Yufereva YM, Pogosova G-N, Oganov RG, et al Depressive symptoms predict death in arterial hypertension and coronary heart disease patients: Results of a 3-year follow-up multicenter study. Eur Heart J 2010;31(Abstract Supplement):376.

[25] Murphy A, Mahal A, Richardson E, et al The economic burden of chronic disease care faced by households in Ukraine: a cross-sectional matching study of angina patients. Int J Equity Health 2013;12:38 10.1186/1475-9276-12-38 23718769 PMC3691525

[26] Murphy A, Jakab M, McKee M, et al Persistent low adherence to hypertension treatment in Kyrgyzstan: how can we understand the role of drug affordability? Health Policy Plan 2016;31:1384–90. 10.1093/heapol/czw080 27315830

[27] Roberts B, Stickley A, Balabanova D, et al The persistence of irregular treatment of hypertension in the former Soviet Union. J Epidemiol Community Health 2012;66:1079–82. 10.1136/jech-2011-200645 22447959

[28] Centre on Global Health Security Working Group on Health Financing. Shared responsibilities for health; a coherent global framework for health financing 2014.

[29] Xu K, Evans D, Carrin G, et al Designing health financing systems to reduce catastrophic health expenditure, 2005.

[30] Bank W World bank development indicators, 2016.

[31] Zaridze D, Maximovitch D, Lazarev A, et al Alcohol poisoning is a main determinant of recent mortality trends in Russia: evidence from a detailed analysis of mortality statistics and autopsies. Int J Epidemiol 2009;38:143–53. 10.1093/ije/dyn160 18775875

[32] Roerecke M, Rehm J Irregular Heavy Drinking Occasions and risk of Ischemic Heart disease: a Systematic review and Meta-Analysis. Am J Epidemiol 2010;171:633–44. 10.1093/aje/kwp451 20142394

[33] Leon DA, Shkolnikov VM, McKee M Alcohol and russian mortality: a continuing crisis. Addiction 2009;104:1630–6. 10.1111/j.1360-0443.2009.02655.x 19681805

[34] Leon DA, Shkolnikov VM, McKee M, et al Alcohol increases circulatory disease mortality in Russia: acute and chronic effects or misattribution of cause? Int J Epidemiol 2010;39:1279–90. 10.1093/ije/dyq102 20591986 PMC2972439

[35] Zaridze D, Lewington S, Boroda A, et al Alcohol and mortality in Russia: prospective observational study of 151?000 adults. The Lancet 2014;383:1465–73. 10.1016/S0140-6736(13)62247-3 PMC4007591 24486187

[36] Roberts B, Gilmore A, Stickley A, et al Changes in Smoking prevalence in 8 countries of the Former Soviet Union between 2001 and 2010. Am J Public Health 2012;102:1320–8. 10.2105/AJPH.2011.300547 22594739 PMC3478012

