# Supplementary material for: Ischaemic heart disease in the former Soviet Union 1990–2015 according to the Global Burden of Disease 2015 Study
Source: Heart. 2017 Sep 7;104(1):58–66. doi: 10.1136/heartjnl-2016-311142 (PMC5749345; doi:10.1136/heartjnl-2016-311142)
Supplement: Supplementary file 1 [file heartjnl-2016-311142supp001.pdf]

Methods Appendix to *Ischemic heart disease in the former Soviet Union, 1990-2015: the Global Burden of Disease 2015 Study*

This appendix provides further methodological detail and supplemental figures and tables.

Supplementary Methods: Tables

Appendix Table 1. Guidelines for Accurate and Transparent Health Estimates Reporting (GATHER) 18-item checklist with description of compliance and location of information for this GBD 2015 Cardiovascular Diseases publication

Appendix Table 2. GBD 2015 CODem model covariates for ischemic heart disease (IHD)

Supplementary Results: Tables & Figures

Appendix Table 2a: Age-standardized IHD DALYs and 95% uncertainty intervals, females, 1990 and 2015, GBD 2015 Study
Appendix Table 2b: Age-standardized IHD DALYs and 95% uncertainty intervals, males, 1990 and 2015, GBD 2015 Study

Appendix Figure 1a: Life expectancy at birth 1990-2015, all sexes, Central Asia, WHO Health for All Database
Appendix Figure 1b: Life expectancy at birth 1990-2015, all sexes, Central Europe, WHO Health for All Database
Appendix Figure 1c: Life expectancy at birth 1990-2015, all sexes, Eastern Europe, WHO Health for All Database

Section 1. GBD 2015 Study Overview

The Global Burden of Diseases, Injuries, and Risk Factors (GBD) enterprise is a systematic, scientific effort to quantify the comparative magnitude of health loss due to diseases, injuries, and risk factors by age, sex, and geography for specific points in time. The GBD construct of the burden of disease is health loss, not income or productivity loss. For decision makers, health-sector leaders, researchers, and informed citizens, the GBD approach provides an opportunity to see the big picture, to compare diseases, injuries, and risk factors, and to understand in a given place, time, and age-sex group what are the most important contributors to health loss.

Measuring disease and injury burden in populations requires a composite metric that captures both premature mortality and the prevalence and severity of ill-health. The 1990 Global Burden of Disease study proposed disability-adjusted life years (DALYs) to measure disease burden. The GBD 2015 Study aimed to calculate disease burden worldwide for 295 causes of death, 310 non-fatal diseases in 21 regions and 195 countries and territories for 1990 and 2015 with methods to enable meaningful comparisons over time.

Glossary: Disability-adjusted life years and Global Burden of Disease definitions[1, 2]

- Disability-adjusted life years (DALYs) are a summary metric of population health. DALYs represent a health gap; they measure the state of a population's health compared to a normative goal. The goal is for individuals to live the standard life expectancy in full health.
- DALYs are the sum of two components: years of life lost due to premature mortality (YLLs) and years lived with disability (YLDs).
- YLLs are computed by multiplying the number of deaths at each age x by a standard life expectancy at age x. The result is the years of life lost due to death before an ideal life expectancy at that age. The standard selected represents the normative goal for survival and has been computed based on the lowest recorded death rates across countries in 2015.
- YLDs are computed as the 2015 prevalence of different disease-sequelae and injury-sequelae multiplied by the disability weight for that sequela. Disability weights are selected on the basis of surveys of the general population about the loss of health associated with the health state related to the disease sequela.
- DALYs are an absolute measure of health loss; they count how many years of healthy life are lost due to death and non-fatal illness or impairment. They reflect the number of individuals who are ill or die in each age-sex group and location. Population size and composition influences the number of DALYs in a population.
- The GBD 2015 disease-and-injury-cause list is a hierarchical list of 310 diseases and injuries. At the first level of disaggregation causes are divided into three broad groups: communicable, maternal, neonatal, and nutritional disorders; non-communicable diseases; and injuries. At each level in the hierarchy, the cause list provides a set of mutually exclusive and collectively exhaustive categories.
- Sequelae—in total, we have identified unique 2619 sequelae of the 310 non-fatal diseases and injuries. For example, diabetic neuropathy is a sequela of diabetes mellitus. To avoid

double counting, a sequela can only appear in the cause-sequela list once even if the same outcome might be claimed by more than one disease.
- Health states—across the 2619 sequelae, 235 unique health states were identified. For example, both malaria and hookworm have mild anaemia as a sequela. Mild anaemia is a unique health state. The list of unique health states serves two purposes: (a) to allow assessment of the total burden of some health states such as anaemia across various causes; and (b) to simplify the task of measuring disability weights for sequelae.
- DALYs presented in this study are not age-weighted and are not discounted for time preference. Base case tabulations for the GBD 1990 and GBD 2000 studies used age-weighting and a 3% discount rate.

GATHER statement

This study is in compliance with the Guidelines for Accurate and Transparent Health Estimates Reporting (GATHER) recommendations. The GBD 2015 capstone papers and their respective supplementary documents contain the general methods, data sources, model selection information, performance and limitation information for the GBD 2015 analyses including detailed GATHER documentation[1,2]. Appendix Table 1 contains GATHER compliance information for this publication.

Geographies estimated

Consistent with the protocol of the Global Burden of Disease Study 2015, we estimated incidence and prevalence of cardiovascular diseases from 1980-2015 for 195 countries and territories, for both sexes, and 15 five-year age groups (age group 10-14 through age group 80+). Regions and countries specific to this analysis are described in the main paper.

GBD Cause List

The GBD Cause List is organized into four levels, consisting of a hierarchy that is mutually exclusive and collectively exhaustive. Details on the overall GBD Cause list have been documented elsewhere[1,2].

Data Sources

A complete list of sources used in the GBD 2015 analyses is available from the GBD 2015 Data Input Sources Tool (http://ghdx.healthdata.org/gbd-2015/data-input-sources).

Section 2. Causes of death modeling methods

Mortality estimates for ischemic heart disease (IHD) were generated using a cause of death ensemble model (CODEm). In brief, CODEm relies on models of cause-specific mortality that include temporally and geographically specific data for specific GBD causes of death, plus covariates that are also temporally and geographically specific. The ensemble modeling approach summarizes weighted contributions of several model forms if the ensemble fit is superior to the fit of any one model form. The CODEm methods approach has been described elsewhere[1]. Specific methodologies for mortality estimates for IHD and other cardiovascular diseases (CVDs) were also described in the GBD 2015 Causes of Death Capstone paper and have been appended here for ease of access[1]. The specific approach to the IHD ensemble model is described below. A list of covariates used in CODEm modeling for IHD can be found in Appendix Table 2.

Flow chart legend

The legend for all flowcharts describing the modeling strategy for cause of death estimates is below.

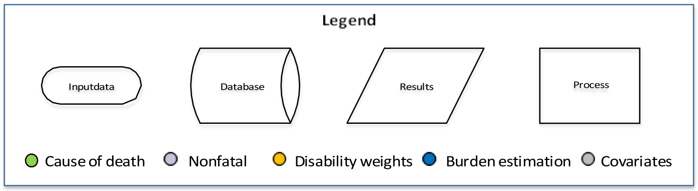

Ischemic Heart Disease

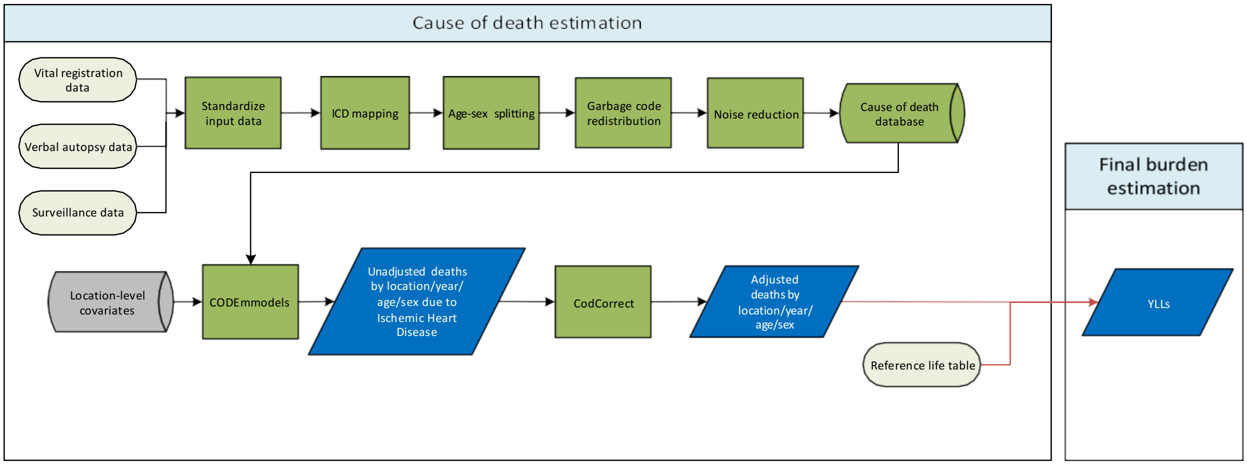

Vital registration, verbal autopsy, and surveillance data were used to model ischemic heart disease. We censored (treated as outliers) verbal autopsy data in countries and subnational locations where high-quality vital registration data were also available. We also censored non-representative subnational verbal autopsy data points, and ICD8 and ICD9 BTL data points that were inconsistent with the rest of the data and created implausible time trends. Finally, we censored data in a number of Indian states identified by experts as poor-quality.

Modeling strategy

We used a standard CODEm approach to model deaths from ischemic heart disease. We tested the validity of each component model with multiple cross-validations, and formed an ensemble multivariate model using the optimally weighted contributions of models with the best individual performance (i.e., lowest root mean squared error). The highest weighted models for ischemic heart disease, and therefore the models that contribute predominantly to estimate results, were spatiotemporal Gaussian process regression (ST-GPR) models of the logit of the cause of death fraction. Cause fractions are applied to population age-sex-location-year-specific population counts to produce age-sex-location-year-specific death rates.

Covariates included in IHD CODEm models are described elsewhere.[3] Since the GBD 2013, wWe have included two new variables in our IHD ensemble model -,: Socio-Demographic Index and the SEV scalar for ischemic heart disease., as possible covariates for selection in the ensemble modeling process. Otherwise, there have been no substantive changes from the CODEm approach used in GBD 2013.

Section 3. Nonfatal IHD modeling methods

IHD morbidity was modeled using a unique software application, Disease Modeling-Metaregression (DisMod-MR) 2.1 platform. DisMod-MR is the latest iteration of a generic disease modelling system but redesigned as a Bayesian meta-regression tool . The Bayesian approach is one of several interpretations of statistical probability in which existing data is used to inform the probability of a given hypothesis i.e. the data is considered as fixed and the hypothesis as random. A meta-regression can be understood as an extension of a meta-analysis whereby data from different studies are pooled into a weighted average, adjusting for sources of variability between studies. DisMod-MR has the capability to combine epidemiological data from multiple sources, reconcile data that are inconsistent and estimate data for regions and parameters with no or little data. It applies a negative-binomial model of disease prevalence, incidence, remission, and case-fatality rates and fits models with a randomized Markov-Chain Monte Carlo algorithm. Non-fatal burden estimates for all disorders in GBD 2010 were calculated using DisMod-MR. Nonfatal estimation and modeling methods have been documented elsewhere[4].

Flow chart legend

The legend for all flowcharts describing the modeling strategy for nonfatal disease estimates is below.

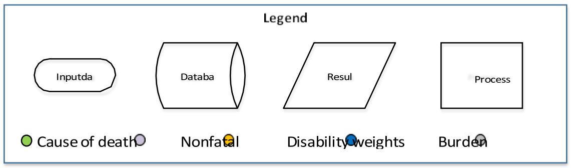

Ischemic Heart Disease

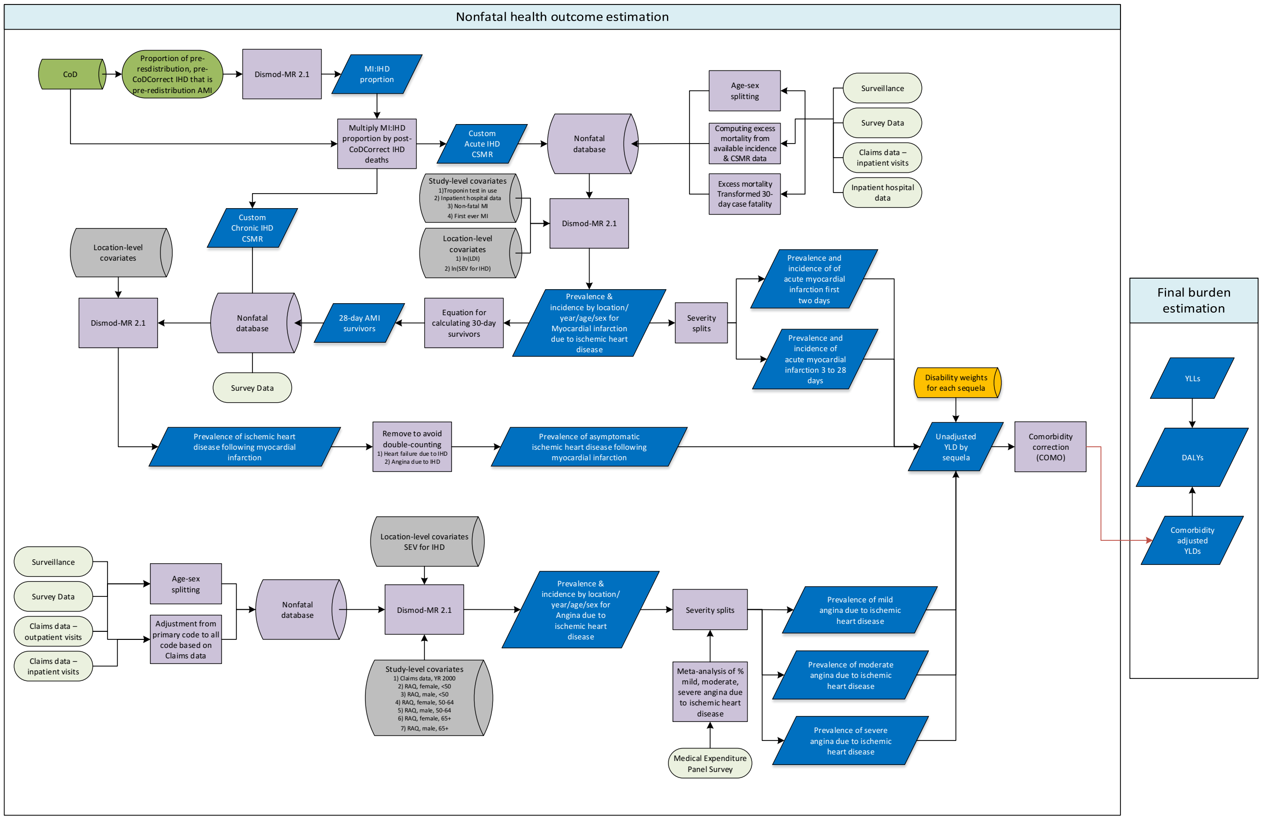

We defined cases of ischemic heart disease using the following definitions:

1) Acute myocardial infarction (AMI): Definite and possible AMI according to the third universal definition of myocardial infarction[5]:
    a. When there is clinical evidence of myocardial necrosis in a clinical setting consistent with myocardial ischemia or
    b. Detection of a rise and/or fall of cardiac biomarker values and with at least one of the following: i) symptoms of ischemia, ii) new or presumed new ST-segment-T wave changes or new left bundle branch block, iii) development of pathological Q waves in the ECG, iv) imaging evidence of new loss of viable myocardium or new regional wall motion abnormality, or v) identification of an intracoronary thrombus by angiography or autopsy.
    c. Sudden (abrupt) unexplained cardiac death, involving cardiac arrest or no evidence of a noncoronary cause of death
    d. Prevalent AMI is condiered to last from the onset of the event to 28 days after the event and is divided into an acute phase (0-2 days) and subacute (3-28 days).
2) Chronic IHD
    a. Angina; clinically diagnosed stable exertional angina pectoris or definite angina pectoris according to the Rose Angina Questionnaire[5], physician diagnosis, or taking

nitrate medication for the relief of chest pain.

b. Asymptomatic ischemic heart disease following myocardial infarction; survival to 28 days following incident AMI. The GBD study does not use estimates based on ECG evidence for prior MI, due to its limited specificity and sensitivity[6].

Input data for nonfatal IHD models

Myocardial infarction

A systematic review was done for myocardial infarction for GBD 2015.

The search strings used were extensive; a full list can be found here: J:\WORK\04_epi\01_database\02_data\cvd_ihd\00_documentation\IHD_Search_Strings_GBD2015\IHD_Search_Split.docx.

The dates of the search were 1/1/2009 – 2/3/2015. 38,522 studies were returned; 194 were extracted (this number includes extractions that were done for STEMI/NSTEMI models and revascularization models that are not currently part of the MI modeling process but may be in the future).

A systematic review for myocardial infarction was also done for GBD 2013. The extensive search terms for that review can be found here: https://hub.ihme.washington.edu/pages/viewpage.action?spaceKey=SR&title=GBD+2013+Literature+Review+Documentation

Literature data included: Myocardial infarction

	Prevalence	Incidence	Mortality risk
Studies	0	93	61
Countries/subnationals	0	39	32
GBD world regions	0	8	10

Apart from inpatient hospital and inpatient claims data, we did not include any data from sources other than the literature for myocardial infarction. We excluded data with broad age ranges where it was impossible to obtain more granular data, as these data caused the known age pattern for increased risk of myocardial infarction to be masked in the estimates generated from DisMod.

We corrected inpatient hospital data and claims data to account for the fact that these data sources do not capture the out-of-hospital cardiac arrest deaths which are part of the universal definition of AMI. We also included a covariate to correct for the change in diagnostic criteria to include troponin measurements. This adjustment was applied to data collected before 2000. We also adjusted data points that were not specific about whether it was the first AMI for included subjects, using studies where only first events were included as the reference. We also adjusted estimates from studies that only included non-fatal cases using study-level covariates.

Angina

A systematic review was not performed for GBD 2015. Updates to systematic reviews are performed on an ongoing schedule across all GBD causes; an update for angina will be performed in the next one to two iterations.

A systematic review for angina was done for GBD 2013. The search terms for that are here: (Angina Pectoris/epidemiology[Mesh] OR Angina Pectoris/mortality[Mesh]) AND (prevalence[Title/Abstract] OR incidence[Title/Abstract]) AND ("2010"[Date - Publication] : "3000"[Date - Publication])

Literature data included: Angina

	Prevalence	Incidence	Mortality risk
Studies	72	0	7
Countries/subnationals	73	0	7
GBD world regions	20	0	5

We included survey data (including NHANES and World Health Study questionnaires) which included the Rose angina quesionnaire items. Prevalence of angina was calculated using the standard algorithm to determine whether the Rose angina quesionnaire was positive or negative.

We excluded data with broad age ranges where it was impossible to obtain more granular data, as these data caused the known age pattern for increased risk of angina to be masked in the estimates generated from DisMod.

We included sex- and age group-specific covariates to adjust prevalence data points obtained from the RAQ using the claims data as the reference since the RAQ has been shown to be neither sensitive nor specific.

Severity split inputs

Acute myocardial infarction was split into two severity levels by length of time since the event – days 1 and 2 versus days 3 through 28. Disability weights were established for these two severities using the standard approach for GBD 2015, which used estimates of relative disability from household surveys of lay people in Bangladesh, Indonesia, Peru, Tanzania, the U.S.A., Hungary, Italy, the Netherlands and Sweden, and an international web-based survey of health professionals.

Angina was split into mild, moderate, and severe groups using information from the Medical Expenditure Panel Survey (MEPS). Disability weights for these severities were also established using the standard approach for GBD 2015.

Acute myocardial infarction

Severity level	Lay description	DW (95% CI)
Acute myocardial infarction, days 1-2	Has severe chest pain that becomes worse with any physical activity. The person feels nauseated, short of breath, and very anxious.	0.432 (0.288-0.579)
Acute myocardial infarction, days 3-28	Gets short of breath after heavy physical activity, and tires easily, but has no problems when at rest. The person has to take medication every day and has some anxiety.	0.074 (0.049-0.105)

Angina pectoris

Severity level	Lay description	DW (95% CI)
Mild angina	Has chest pain that occurs with strenuous physical activity, such as running or lifting heavy objects. After a brief rest, the pain goes away.	0.033 (0.02-0.052)
Moderate angina	Has chest pain that occurs with moderate physical activity, such as walking uphill or more than half a kilometer (around a quarter-mile) on level ground. After a brief rest, the pain goes away.	0.08 (0.052-0.113)
Severe angina	Has chest pain that occurs with minimal physical activity, such as walking only a short distance. After a brief rest, the pain goes away. The person avoids most physical activities because of the pain.	0.167 (0.11-0.24)

Modeling strategy

Myocardial infarction

- We first calculated custom cause-specific mortality estimates using data from cause of death data prior to redistributing garbage codes (vague or ambiguous cause of death codes), generating age-sex-country specific proportions of IHD deaths that were due to AMI (acute IHD) vs those due to other causes of IHD (chronic IHD). IHD age-standardized death rate data were then used, along with incidence and excess mortality data and other covariates (table below), in a DisMod model to estimate the prevalence and incidence of myocardial infarction due to ischemic heart disease.
- These estimates were split into prevalence and incidence estimates for days 1-2 and days 3-28 post event. Disability weights were assigned to each of these two groupings.
- We set a value prior of one month for remission (11/13) from the AMI health state. We also set a value prior for the maximum excess mortality rate of 10 for all ages. We included log-transfomed lag distributed income per capita (lnLDI) ss a fixed-effect country-level covariate on excess mortality, forcing an inverse relationship.

Study covariate	Parameter	Beta	Exponentiated beta
Diagnostic blood sample (troponin)	incidence	-.4432(-.4543 to -.44)	.6419 (.6349 to .644)
Hospital data	incidence	-1.5e-04 (-4.4e-04 to -4.1e-05)	.9999(.9996 to 1)
First ever MI	incidence	-.002(-.009 to -5.1e-05)	.998(.9911 to .9999)
Non fatal MI	incidence	-8.9e-04(-.0024 to -2.3e-04)	.9991(.9976 to .9998)
lag distributed income (international $ per capita)	excess mortality rate	-.1005(-.1027 to -.1)	.9044(.9024 to .9048)

Asymptomatic ischemic heart disease

- Excess mortality estimates from the myocardial infarction model were used to generate data of the incidence of surviving 28 days post-event.
- We used these data, along with the estimates of CSMR due to chronic IHD (the other part of the proportion described in step 1) and excess mortality data in a DisMod model to estimate the prevalence of persons with IHD following myocardial infarction. This estimate included subjects with angina and heart failure; a proportion of this prevalence was removed in order to avoid double counting based on evidence from the literature (2). The result of this step generates estimates of asymptomatic ischemic heart disease following myocardial infarction.
- We set a value prior of 0 for remission for all ages.

Study covariate	Parameter	Beta	Exponentiated beta
Log-transformed age-standardized SEV scalar: IHD	incidence	.9319(.9187 to .9452)	2.539(2.506 to 2.573)

Angina

We used prevalence data from the literature and USA claims databases, along with data on mortality risk to estimate the prevalence and incidence of angina for all locations.

- o The proportion of mild, moderate, and severe angina was determined by the standard approach for severity splitting for GBD 201 (described above).
- o We included a value prior of 0 for remission for all ages. We also included a value prior of 1 for excess mortality for all ages.
- o We included age- and sex-specific study-level covariates to adjust data points based on RAQ, using data points from the claims database as the reference.
- o We also included the log-transformed, age-standardized SEV scalar for IHD as a fixed effect country-level covariate.

Study covariate	Parameter	Beta	Exponentiated beta
RAQ, female, less than 50	prevalence	2.435(2.326 - 2.497)	11.42(10.24 - 12.15)
RAQ, male, less than 50	prevalence	.9454(.9349 - .9499)	2.574(2.547 - 2.585)
RAQ, female, 50 to 64	prevalence	1.484(1.447 - 1.5)	4.411(4.25 - 4.482)
RAQ, male, 50 to 64	prevalence	.9897(.9606 - .9997)	2.69(2.613 - 2.717)
RAQ, female, 65 plus	prevalence	.2929(.2719 - .2998)	1.34(1.312 - 1.35)
RAQ, male, 65 plus	prevalence	.2891(.2582 - .2997)	1.335(1.295 - 1.349)
Log-transformed age-standardized SEV scalar: IHD	prevalence	1.238(1.209 - 1.249)	3.449(3.35 - 3.487)

Apart from inclusion of hospital data and claims data, there have been no substantive changes in the modeling strategy for myocardial infarction, asymptomatic ischemic heart disease following myocardial infarction, and angina from GBD 2013.

Supplementary Methods References

1. GBD Mortality and Causes of Death Collaborators. Global, regional, and national life expectancy, all-cause and cause-specific mortality for 249 causes of death, 1980–2015: a systematic analysis for the Global Burden of Disease Study 2015. *The Lancet* 2016;388:1459–1544.

2. GBD 2015 Disease and Injury Incidence and Prevalence Collaborators. Global, regional, and national incidence, prevalence, and years lived with disability for 310 diseases and injuries, 1990-2015: a systematic analysis for the Global Burden of Disease Study 2015. *The Lancet* 2016; 388:1603–1658
3. Moran AE, Forouzanfar MH, Roth GA, Mensah GA, Ezzati M, Flaxman A, Murray CJ, Naghavi M. The global burden of ischemic heart disease in 1990 and 2010: The global burden of disease 2010 study. *Circulation*. 2014;129:1493-1501

4. Reményi, B. et al. Nat. Rev. Cardiol. 9, 297–309 (2012); published online 28 February 2012

5. Third Universal Definition of Myocardial Infarction
Kristian Thygesen, Joseph S. Alpert, Allan S. Jaffe, Maarten L. Simoons, Bernard R. Chaitman and

Harvey D. White and the Writing Group on behalf of the Joint ESC/ACCF/AHA/WHF Task Force for the Universal Definition of Myocardial Infarction
Circulation. 2012;126:2020-2035, originally published October 15, 2012

6. Rose G, McCartney P, Reid DD. Self-administration of a questionnaire on chest pain and intermittent claudication. Br J Prev Soc Med. 1977 Mar;31(1):42–48. [PMC free article] [PubMed]

7. Heart. 2012 Nov;98(22):1660-6. doi: 10.1136/heartjnl-2012-302318. Epub 2012 Sep 2. Utility of self-reported diagnosis and electrocardiogram Q-waves for estimating myocardial infarction prevalence: an international comparison study. Moran A., Shen A, Turner-Lloveras D, Khan A, Clays E, Palmas W, De Bacquer D.

8. Hatano S. Experience from a multicentre stroke register: a preliminary report. Bulletin of the World Health Organisation 1976;54(5):541–553.

Appendix Table 1. GATHER checklist of information that should be included in reports of global health estimates, with description of compliance and location of information for Global, regional, and national prevalence, mortality, years of life lost, years lived with disability, and disability-adjusted life years for 11 Causes of Cardiovascular Disease, 1990 to 2015 – a systematic analysis for the Global Burden of Disease Study 2015.

#	GATHER checklist item	Description of compliance	Reference
**Objectives and funding**			
1	Define the indicators, populations, and time periods for which estimates were made.	Narrative provided in paper and appendix describing indicators, definitions, and populations.	Manuscript; Methods Appendix, Section 1. GBD Overview
2	List the funding sources for the work.	Funding sources listed at end of paper.	Funding Sources
**Data Inputs**			
For all data inputs from multiple sources that are synthesized as part of the study:			
3	Describe how the data were identified and how the data were accessed.	Narrative description of data seeking methodology provided in previously published appendices.	1) GBD 2015 Mortality and Causes of Death Collaborators. Global, regional, and national life expectancy, all-cause mortality, and cause-specific mortality for 249 causes of death, 1980-2015: a systematic analysis for the Global Burden of Disease Study 2015. The Lancet 2016; 388:1459–1544.<br>2) GBD 2015 Disease and Injury Incidence and Prevalence Collaborators. Global, regional, and national incidence, prevalence, and years lived with disability for 310 diseases and injuries, 1990-2015: a systematic analysis for the Global Burden of Disease Study 2015. The Lancet 2016; 388:1603–1658
4	Specify the inclusion and exclusion criteria. Identify all ad-hoc exclusions.	Narrative about inclusion and exclusion criteria by data type provided in previously published appendices.	1) GBD 2015 Mortality and Causes of Death Collaborators. Global, regional, and national life expectancy, all-cause mortality, and cause-specific mortality for 249 causes of death, 1980-2015: a systematic analysis for the Global Burden of Disease Study 2015. The Lancet 2016; 388:1459–1544.<br>2) GBD 2015 Disease and Injury Incidence and Prevalence Collaborators. Global, regional, and national incidence, prevalence, and years lived with disability for 310 diseases and injuries, 1990-2015: a systematic

			analysis for the Global Burden of Disease Study 2015. The Lancet 2016; 388:1603–1658
5	Provide information on all included data sources and their main characteristics. For each data source used, report reference information or contact name/institution, population represented, data collection method, year(s) of data collection, sex and age range, diagnostic criteria or measurement method, and sample size, as relevant.	Interactive, online data source tool that provides metadata for data sources by component, geography, cause, risk, or impairment has been developed.	Online data tools: http://ghdx.healthdata.org/gbd-2015/data-input-sources
6	Identify and describe any categories of input data that have potentially important biases (e.g., based on characteristics listed in item 5).	Summary of known biases by cause included in methodological approaches sections of previously published appendices.	1) GBD 2015 Mortality and Causes of Death Collaborators. Global, regional, and national life expectancy, all-cause mortality, and cause-specific mortality for 249 causes of death, 1980-2015: a systematic analysis for the Global Burden of Disease Study 2015. The Lancet 2016; 388:1459–1544.<br>2) GBD 2015 Disease and Injury Incidence and Prevalence Collaborators. Global, regional, and national incidence, prevalence, and years lived with disability for 310 diseases and injuries, 1990-2015: a systematic analysis for the Global Burden of Disease Study 2015. The Lancet 2016; 388:1603–1658
For data inputs that contribute to the analysis but were not synthesized as part of the study:			
7	Describe and give sources for any other data inputs.	Included in list of all data sources provided on online data source tool.	Online data tools http://ghdx.healthdata.org/gbd-2015/data-input-sources
For all data inputs:			
8	Provide all data inputs in a file format from which data can be efficiently extracted (e.g., a spreadsheet as opposed to a PDF), including all relevant meta-data listed in item 5. For any data inputs that cannot be shared due to ethical or legal reasons, such as third-party ownership, provide a contact name or the name of the institution that retains the right to the data.	Downloads of input data will be available through online tools, including data visualization tools and data query tools. Input data not available in tools will be made available upon request.	Online data tools http://www.healthdata.org/results/data-visualizations; http://ghdx.healthdata.org/; http://ghdx.healthdata.org/gbd-data-tool
**Data analysis**			
9	Provide a conceptual overview of the data analysis method. A diagram may be helpful.	Flow diagrams of the overall methodological processes, as well as cause-specific modelling processes have been provided.	Methods Appendix, Section 2. Causes of death modelling methods; Methods Appendix, Section 3. Nonfatal modelling methods
10	Provide a detailed description of all steps of the analysis, including mathematical formulae. This description should cover, as relevant, data	Detailed descriptions of all steps of the analysis were included in the methodological	1) GBD 2015 Mortality and Causes of Death Collaborators. Global, regional, and national

	cleaning, data pre-processing, data adjustments and weighting of data sources, and mathematical or statistical model(s).	approaches sections of previously published appendices.	life expectancy, all-cause mortality, and cause-specific mortality for 249 causes of death, 1980-2015: a systematic analysis for the Global Burden of Disease Study 2015. The Lancet 2016; 388:1459–1544.<br>2) GBD 2015 Disease and Injury Incidence and Prevalence Collaborators. Global, regional, and national incidence, prevalence, and years lived with disability for 310 diseases and injuries, 1990-2015: a systematic analysis for the Global Burden of Disease Study 2015. The Lancet 2016; 388:1603–1658
11	Describe how candidate models were evaluated and how the final model(s) were selected.	Provided in the methodological write-ups of previously published appendices.	1) GBD 2015 Mortality and Causes of Death Collaborators. Global, regional, and national life expectancy, all-cause mortality, and cause-specific mortality for 249 causes of death, 1980-2015: a systematic analysis for the Global Burden of Disease Study 2015. The Lancet 2016; 388:1459–1544.<br>2) GBD 2015 Disease and Injury Incidence and Prevalence Collaborators. Global, regional, and national incidence, prevalence, and years lived with disability for 310 diseases and injuries, 1990-2015: a systematic analysis for the Global Burden of Disease Study 2015. The Lancet 2016; 388:1603–1658
12	Provide the results of an evaluation of model performance, if done, as well as the results of any relevant sensitivity analysis.	Provided in the methodological write-ups of previously published appendices.	1) GBD 2015 Mortality and Causes of Death Collaborators. Global, regional, and national life expectancy, all-cause mortality, and cause-specific mortality for 249 causes of death, 1980-2015: a systematic analysis for the Global Burden of Disease Study 2015. The Lancet 2016; 388:1459–1544.<br>2) GBD 2015 Disease and Injury Incidence and Prevalence Collaborators. Global, regional,

			and national incidence, prevalence, and years lived with disability for 310 diseases and injuries, 1990-2015: a systematic analysis for the Global Burden of Disease Study 2015. The Lancet 2016; 388:1603–1658
13	Describe methods for calculating uncertainty of the estimates. State which sources of uncertainty were, and were not, accounted for in the uncertainty analysis.	Provided in the methodological write-ups of previously published appendices.	1) GBD 2015 Mortality and Causes of Death Collaborators. Global, regional, and national life expectancy, all-cause mortality, and cause-specific mortality for 249 causes of death, 1980-2015: a systematic analysis for the Global Burden of Disease Study 2015. The Lancet 2016; 388:1459–1544.<br>2) GBD 2015 Disease and Injury Incidence and Prevalence Collaborators. Global, regional, and national incidence, prevalence, and years lived with disability for 310 diseases and injuries, 1990-2015: a systematic analysis for the Global Burden of Disease Study 2015. The Lancet 2016; 388:1603–1658
14	State how analytic or statistical source code used to generate estimates can be accessed.	Access statement provided.	http://ghdx.healthdata.org/global-burden-disease-study-2015
**Results and Discussion**			
15	Provide published estimates in a file format from which data can be efficiently extracted.	GBD 2015 results are available through online data visualization tools, the Global Health Data Exchange, and the online data query tool (these tools are already available for GBD 2013 results).	Online data tools http://www.healthdata.org/results/data-visualizations; http://ghdx.healthdata.org/; http://ghdx.healthdata.org/gbd-data-tool
16	Report a quantitative measure of the uncertainty of the estimates (e.g. uncertainty intervals).	Uncertainty intervals are provided with all results.	Main text; Online data tools http://www.healthdata.org/results/data-visualizations; http://ghdx.healthdata.org/; http://ghdx.healthdata.org/gbd-data-tool
17	Interpret results in light of existing evidence. If updating a previous set of estimates, describe the reasons for changes in estimates.	Discussion of methodological changes between GBD rounds provided in the appendix.	Methods Appendix, Section 2. Causes of death modelling methods; Methods Appendix, Section 3. Nonfatal modelling methods

18	Discuss limitations of the estimates. Include a discussion of any modelling assumptions or data limitations that affect interpretation of the estimates.	Discussion of limitations provided in the narrative of the main paper as well as in the methodological write-ups of previously published appendices	1) Main text, Limitations<br>2) GBD 2015 Mortality and Causes of Death Collaborators. Global, regional, and national life expectancy, all-cause mortality, and cause-specific mortality for 249 causes of death, 1980-2015: a systematic analysis for the Global Burden of Disease Study 2015. The Lancet 2016; 388:1459–1544.<br>3) GBD 2015 Disease and Injury Incidence and Prevalence Collaborators. Global, regional, and national incidence, prevalence, and years lived with disability for 310 diseases and injuries, 1990-2015: a systematic analysis for the Global Burden of Disease Study 2015. The Lancet 2016; 388:1603–1658

Appendix Table 2. GBD 2015 CODem model covariates used for IHD mortality estimation

Cause Name	Level	Covariate Name	Direction
Ischemic heart disease	2	Alcohol (liters per capita)	0
Ischemic heart disease	2	Animal Fats (kcal per capita)	1
Ischemic heart disease	1	Cholesterol (total, mean per capita)	1
Ischemic heart disease	1	Elevation Under 100m (proportion)	1
Ischemic heart disease	3	LDI (I$ per capita)	-1
Ischemic heart disease	1	Log-transformed SEV scalar: IHD	1
Ischemic heart disease	2	Omega 3 Adjusted(g)	-1
Ischemic heart disease	3	Pulses Legumes Adjusted(g)	-1
Ischemic heart disease	3	Red Meats Adjusted(g)	1
Ischemic heart disease	1	Smoking Prevalence	1
Ischemic heart disease	3	Sociodemographic Status	0
Ischemic heart disease	2	Vegetables Adjusted(g)	-1

Appendix Table 3. Age-standardized IHD DALYs and 95% uncertainty intervals, females, 1990 and 2015, GBD 2015 Study

**Year**	1990		2015	
**Summary**	Mean	95% CI	Mean	95% CI
**Location**				
**Central Asia**				
Armenia	3988.229	(3,729.14 to 4,249.37)	2628.782	(2,403.1 to 2,861.02)
Azerbaijan	6207.38	(5,914.03 to 6,472.82)	3852.242	(3,559.3 to 4,147.08)
Georgia	4791.103	(4,478.55 to 5,105.38)	2610.241	(2,380.93 to 2,868.4)
Kazakhstan	3980.195	(3,850.27 to 4,109.32)	3682.846	(3,354.07 to 4,032.9)
Kyrgyzstan	3955.935	(3,720.01 to 4,203.71)	4244.966	(3,948.91 to 4,535.88)
Mongolia	5112.037	(4,573.72 to 5,696.53)	3893.987	(3,503.63 to 4,435.6)
Tajikistan	4595.53	(4,299.24 to 4,883.76)	3873.171	(3,559.55 to 4,219.6)
Turkmenistan	6017.777	(5,830.56 to 6,197.2)	4854.004	(4,623.78 to 5,103.51)
Uzbekistan	4965.375	(4,628.45 to 5,352.19)	4283.739	(3,833.78 to 4,656.56)
**Central Europe**				
Albania	1862.804	(1,746.42 to 1,997.29)	1731.696	(1,570.61 to 1,882.58)
Bosnia and Herzegovina	2829.701	(2,654.01 to 2,989.66)	1222.103	(1,103.43 to 1,347.02)
Bulgaria	3749.422	(3,639.38 to 3,862.16)	2440.872	(2,292.73 to 2,593.38)
Croatia	2554.918	(2,451.36 to 2,657.26)	1650.136	(1,556.45 to 1,754.93)
Czech Republic	3613.426	(3,497.45 to 3,730.38)	1675.764	(1,596.54 to 1,754.8)
Hungary	3389.073	(3,285.66 to 3,494.49)	1934.747	(1,824.62 to 2,051.46)
Macedonia	2473.328	(2,325.91 to 2,619.92)	1718.974	(1,577.0 to 1,860.25)
Montenegro	1880.676	(1,684.59 to 2,094.82)	1610.996	(1,453.52 to 1,780.11)
Poland	3698.745	(3,592.13 to 3,804.8)	1445.372	(1,370.3 to 1,526.05)
Romania	3511.412	(3,400.39 to 3,619.36)	2118.499	(2,000.53 to 2,251.8)
Serbia	2740.445	(2,551.79 to 2,968.95)	1862.266	(1,749.32 to 1,977.04)
Slovakia	4219.644	(4,055.57 to 4,367.95)	2004.094	(1,901.6 to 2,106.13)
Slovenia	1696.715	(1,612.74 to 1,781.76)	743.362	(681.88 to 804.5)
**Eastern Europe**				

Belarus	4226.691	(4,053.55 to 4,385.05)	4154.211	(3,903.64 to 4,404.66)
Estonia	3958.618	(3,836.05 to 4,083.13)	1715.818	(1,593.48 to 1,841.33)
Latvia	3982.517	(3,858.64 to 4,113.85)	2241.326	(2,122.35 to 2,364.34)
Lithuania	3940.141	(3,832.37 to 4,048.84)	2183.028	(2,086.71 to 2,280.63)
Moldova	5306.786	(5,161.83 to 5,446.43)	3490.841	(3,335.17 to 3,642.84)
Russia	3767.379	(3,650.28 to 3,873.63)	3118.89	(2,974.35 to 3,279.01)
Ukraine	4330.483	(4,210.4 to 4,437.87)	4619.934	(4,381.95 to 4,845.87)

Appendix Table 4. Age-standardized IHD DALYs and 95% uncertainty intervals, males, 1990 and 2015, GBD 2015 Study

**Year**	1990		2015	
**Summary**	Mean	95% UI	Mean	95% UI
**Location**				
**Central Asia**				
Armenia	7509.222	(7,119.85 to 7,910.62)	6201.984	(5,780.67 to 6,634.96)
Azerbaijan	11135.963	(10,565.04 to 11,704.36)	7449.151	(6,720.74 to 8,322.97)
Georgia	9351.756	(8,648.03 to 10,094.41)	7154.733	(6,447.35 to 7,787.87)
Kazakhstan	8194.993	(7,947.35 to 8,417.95)	8113.458	(7,385.26 to 8,840.39)
Kyrgyzstan	7239.21	(6,836.16 to 7,662.11)	8092.838	(7,535.05 to 8,793.91)
Mongolia	6761.623	(5,457.69 to 7,377.76)	7723.678	(7,060.52 to 8,374.61)
Tajikistan	6858.244	(6,371.98 to 7,654.73)	5680.84	(5,052.38 to 6,650.98)
Turkmenistan	10488.31	(9,626.23 to 10,777.45)	9237.98	(8,751.44 to 9,697.65)
Uzbekistan	7868.657	(7,377.06 to 8,457.64)	7511.248	(6,963.36 to 8,124.78)
**Central Europe**				
Albania	4001.211	(3,746.22 to 4,237.78)	3752.264	(3,291.09 to 4,203.79)
Bosnia and Herzegovina	5625.66	(5,265.76 to 5,952.92)	2571.687	(2,354.9 to 2,872.69)
Bulgaria	7049.735	(6,841.76 to 7,227.78)	5451.304	(5,183.15 to 5,740.47)
Croatia	5667.204	(5,480.29 to 5,892.01)	3299.31	(3,136.46 to 3,469.96)
Czech Republic	8293.643	(8,074.4 to 8,497.05)	3464.244	(3,328.8 to 3,610.99)
Hungary	7735.591	(7,522.89 to 7,974.19)	4038.884	(3,776.62 to 4,281.55)
Macedonia	4864.985	(4,601.18 to 5,118.82)	3631.745	(3,329.63 to 3,921.1)
Montenegro	4427.549	(4,050.03 to 4,819.1)	4064.1	(3,661.59 to 4,439.93)
Poland	9157.149	(8,940.54 to 9,373.56)	3746.673	(3,591.68 to 3,896.44)
Romania	6429.242	(6,238.25 to 6,592.67)	4550.267	(4,309.15 to 4,797.0)
Serbia	4987.439	(4,587.23 to 5,330.01)	3581.611	(3,336.45 to 3,783.96)
Slovakia	9130.585	(8,796.6 to 9,483.97)	4205.574	(3,944.39 to 4,429.09)
Slovenia	4011.739	(3,837.95 to 4,200.69)	1718.037	(1,559.05 to 1,861.76)

**Eastern Europe**				
Belarus	8831.45	(8,439.76 to 9,200.38)	10683.431	(9,909.94 to 11,373.8)
Estonia	9405.983	(9,137.24 to 9,670.59)	4041.759	(3,741.34 to 4,475.62)
Latvia	9460.63	(9,218.81 to 9,712.65)	5688.401	(5,423.08 to 5,995.83)
Lithuania	8538.865	(8,330.14 to 8,754.86)	5859.117	(5,625.39 to 6,164.56)
Moldova	7993.08	(7,776.75 to 8,190.72)	6374.971	(6,127.38 to 6,622.1)
Russia	8581.682	(8,315.52 to 8,846.71)	7772.592	(7,461.94 to 8,077.67)
Ukraine	8341.21	(8,097.06 to 8,567.61)	9614.887	(9,181.6 to 10,040.12)

Appendix Figure 1a: Life expectancy at birth 1990-20, both sexes, Central Asia (WHO Health for All Database[1])

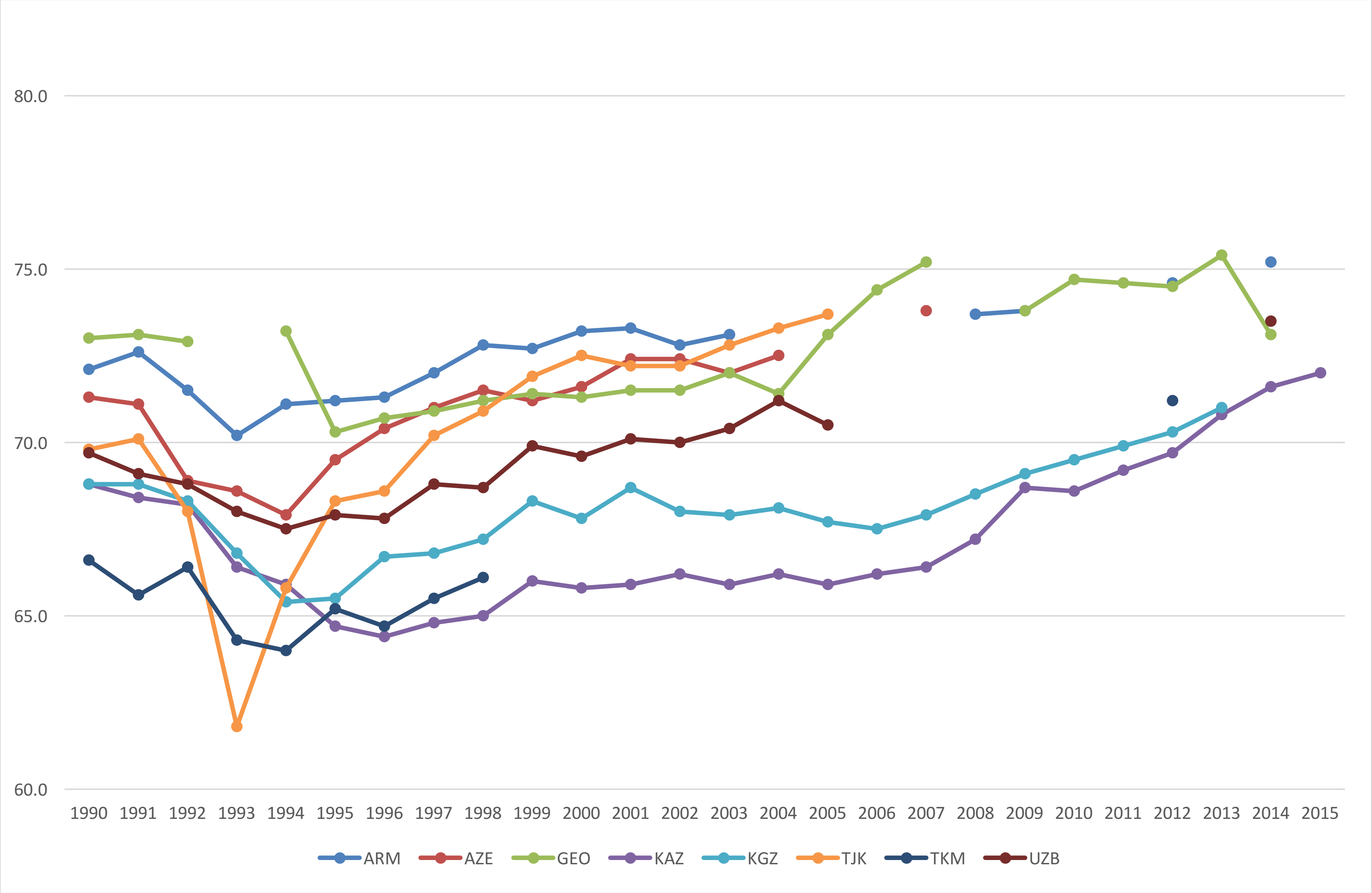

Appendix Figure 1a: Life expectancy at birth 1990-2015, both sexes, Central Europe (WHO Health for All Database[1])

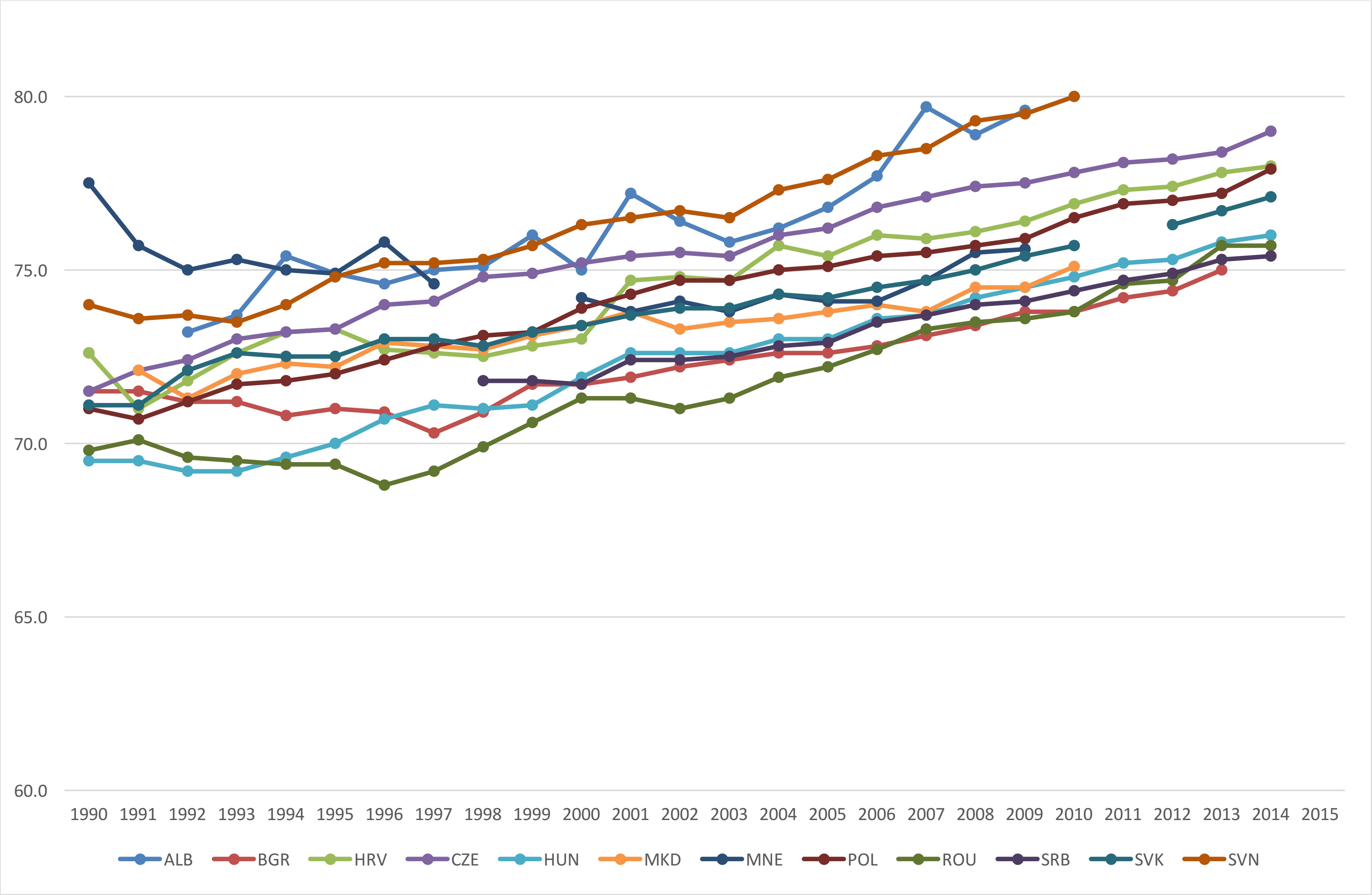

Appendix Figure 1a: Life expectancy at birth 1990-2015, both sexes, Eastern Europe (WHO Health for All Database[1])

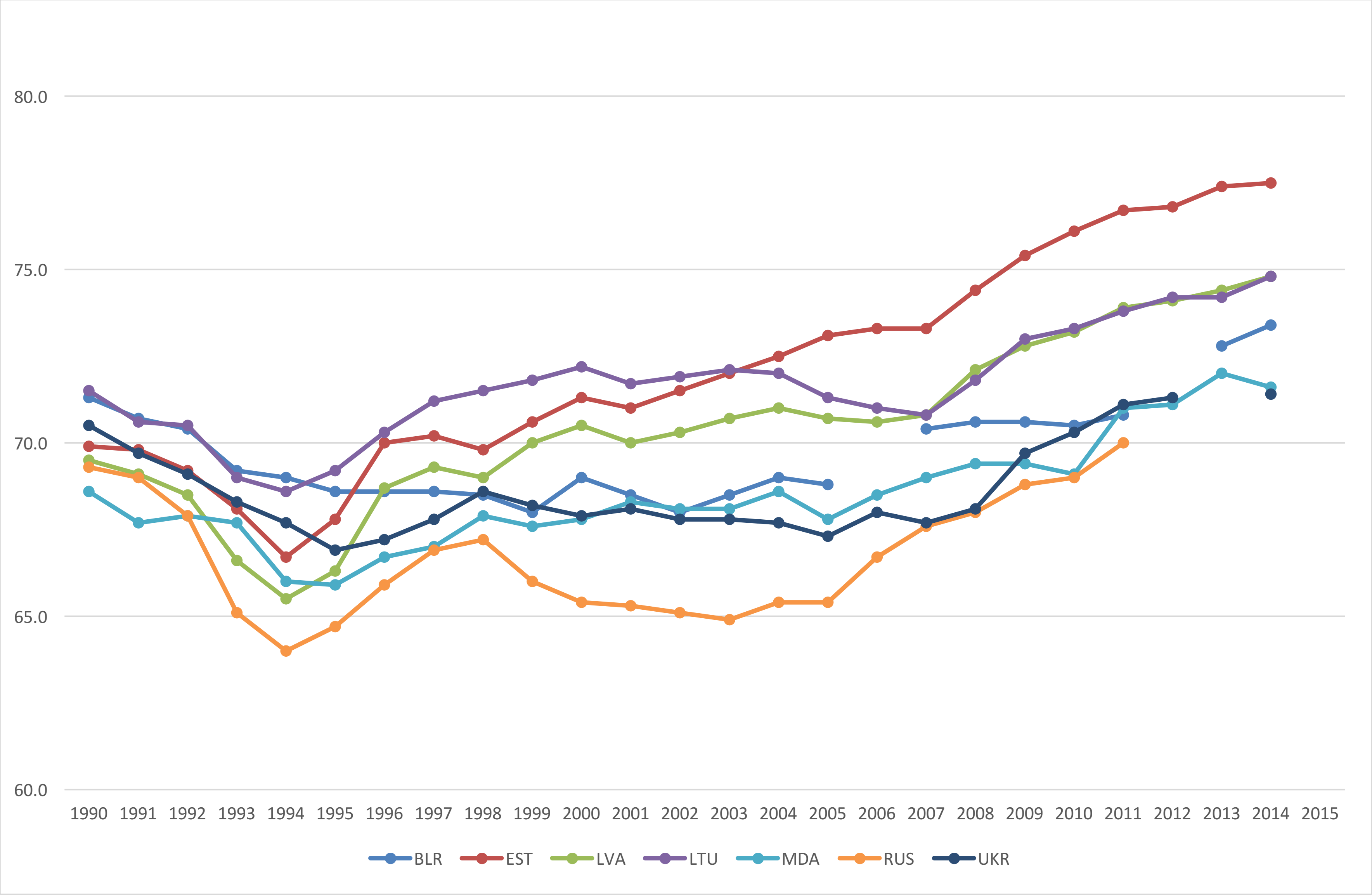

1. World Health Organization European Health for All Databse. http://data.euro.who.int/hfadb/. Accessed April 1, 2017.
